# Beyond Bees: Evidence of Bird Visitation and Putative Pollination in the Golden Lotus (*Musella lasiocarpa*)—One of the Six Buddhist Flowers—Through Field Surveys and Citizen Science

**DOI:** 10.3390/plants14203157

**Published:** 2025-10-14

**Authors:** Sinzinando Albuquerque-Lima, Bruno Henrique dos Santos Ferreira, André Rodrigo Rech, Jeff Ollerton, Klaus Lunau, Guy Smagghe, Kai-Qin Li, Paulo Eugênio Oliveira, Zong-Xin Ren

**Affiliations:** 1 Key Laboratory for Plant Diversity and Biogeography of East Asia, Kunming Institute of Botany, Chinese Academy of Sciences, Kunming 650201, China; brubiologia19@hotmail.com (B.H.d.S.F.); andrerodrigorech@gmail.com (A.R.R.); jeff.ollerton@gmail.com (J.O.); 2 Instituto de Biodiversidade e Sustentabilidade–NUPEM, Universidade Federal do Rio de Janeiro, Av. Amaro Reinaldo dos Santos Silva, 764, São José do Barreto, Macaé 27965-045, RJ, Brazil; 3 Programa de Pós-Graduação em Ecologia e Conservação, Instituto de Biociências, Universidade Federal de Mato Grosso do Sul, Campo Grande 79070-900, MS, Brazil; 4 Departamento de Engenharia Florestal, Universidade Federal dos Vales do Jequitinhonha e Mucuri (UFVJM), Campus JK, Diamantina 39100-000, MG, Brazil; 5 Faculty of Arts, Science and Technology, University of Northampton, Waterside Campus, Northampton NN1 5PH, UK; 6 Faculty of Mathematics and Natural Sciences, Institute of Sensory Ecology, Heinrich Heine University Düsseldorf, 40225 Düsseldorf, Germany; klaus.lunau@hhu.de; 7 Institute of Entomology, Guizhou University, Guiyang 550025, China; guysma9@gmail.com; 8 Department of Biology, Vrije Universiteit Brussel (VUB), 1050 Brussels, Belgium; 9 Department of Plants and Crops, Ghent University, 9000 Ghent, Belgium; 10 Kunming Natural History Museum of Zoology, Kunming Institute of Zoology, Chinese Academy of Sciences, Kunming 650201, China; likaiqin@mail.kiz.ac.cn; 11 Instituto de Biologia, Universidade Federal de Uberlândia, Campus Umuarama, Uberlândia 38402-018, MG, Brazil; poliveiragm@gmail.com

**Keywords:** citizen science, cultural significance, insect pollination, nectar, sunbirds, flower color, flower-visiting birds, Yunnan

## Abstract

Vertebrate pollination is widespread in Musaceae, with birds and bats serving as the main pollen vectors across the family. While these systems are typically well defined, the Golden Lotus (*Musella lasiocarpa*) has long been regarded as an exception, presumed to rely on insect, particularly bee, pollination. In this study, we challenge that assumption by providing the first comprehensive evidence of bird visitation and putative pollination in *M. lasiocarpa*. Through field surveys complemented by citizen science observations, we documented an unexpectedly rich assemblage of avian visitors: twelve bird species from five families regularly foraged at flowers and likely acted as pollinators. This represents a striking expansion of the known potential pollinator spectrum for the species and highlights a previously overlooked dimension of its reproductive ecology. The floral traits of *M. lasiocarpa*, including vivid bracts, accessible nectar, and extended flowering, align closely with adaptations to bird pollination. Beyond clarifying the natural history of the Golden Lotus, our findings reveal broader insights into the ecological and cultural significance of bird pollination in the Chinese flora, with implications for both biodiversity conservation and horticultural practices.

## 1. Introduction

The relationship between birds and flowers has a long evolutionary history, encompassing diverse ecological interactions such as florivory, nectar robbery, pollen feeding, and pollination. Approximately 1400 bird species, belonging to 74 families across 11 orders, are known or suspected to participate in the pollination of nearly 20,000 flowering plant species worldwide [1]. Bird pollination is especially prominent in certain regions, with hummingbirds (Trochilidae) serving as key pollinators in the Americas and sunbirds (Nectariniidae) fulfilling a similar role in Africa and Asia [2]. Despite this remarkable diversity, pollination by birds remains an important but comparatively underexplored ecological interaction in Asia [3].

Among the plant families that are frequently involved in bird pollination, the banana family (Musaceae) stands out. Native to tropical Africa and Asia, Musaceae holds exceptional ethnobotanical, horticultural, nutritional, and ecological importance [4]. The family comprises about 80 species distributed across three genera, *Ensete* Bruce ex Horan., *Musa* L., and *Musella* (Franch.) C.Y. Wu ex H.W. Li [5], and includes bananas and plantains, which form a major component of the global food base. In addition to their role as staple crops, Musaceae species also provide critical resources for diverse animals, including both pollinators and seed dispersers [4,6,7,8,9,10].

**Figure 1:**
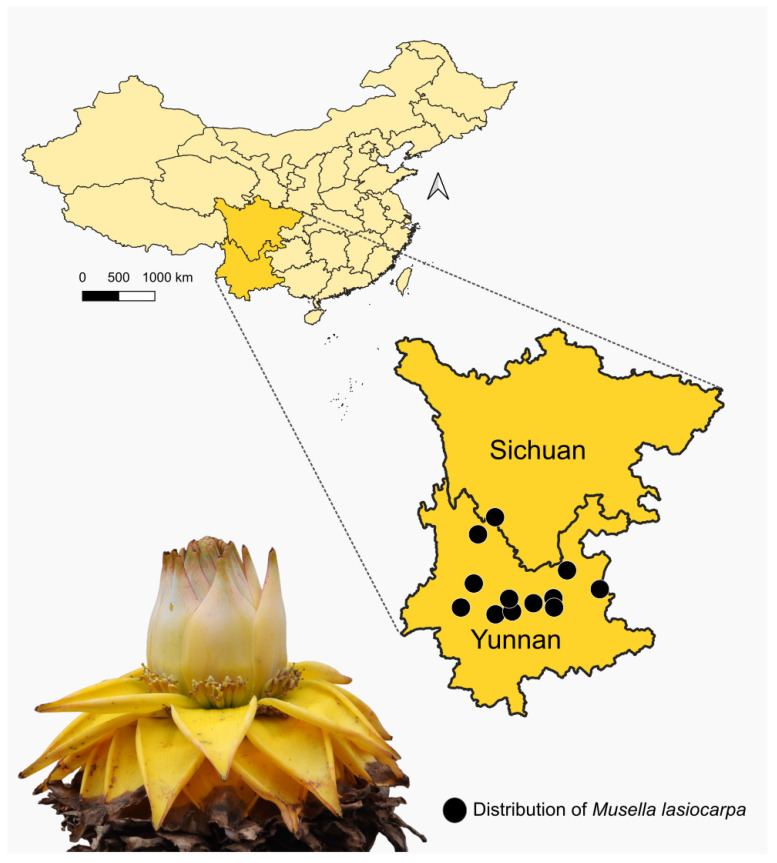
Distribution map of *Musella lasiocarpa* (Franch.) C.Y. Wu ex H.W. Li (Musaceae), based on data available from GBIF [14]. The Golden Lotus is an emblematic herbaceous plant native to south-western China, particularly in the provinces of Sichuan and Yunnan. Photo credit: Sinzinando Albuquerque-Lima.

**Figure 2:**
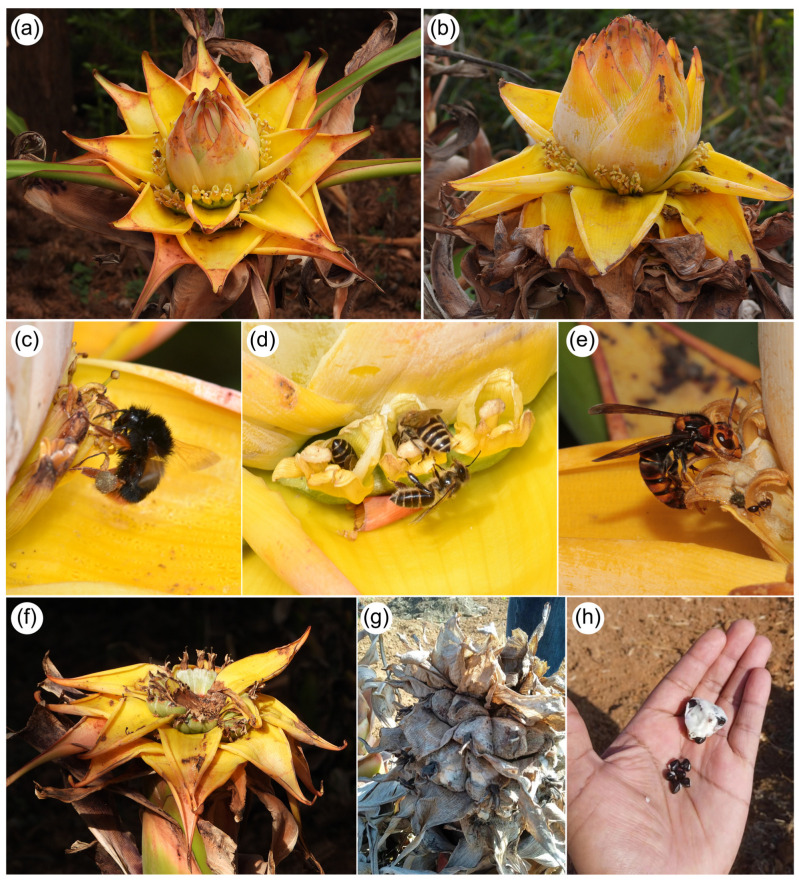
Floral biology of the Golden Lotus (*M. lasiocarpa*). (**a**)—Inflorescence in the female phase. (**b**)—Inflorescence in the male phase. (**c**)—Bumblebee (*Bombus* [*Pyrobombus*] *flavescens* Smith, 1852) visiting male flowers. (**d**)—Female flowers visited by honeybees (*Apis cerana* Fabricius, 1793). (**e**)—*Vespa velutina* visiting male flowers. (**f**–**h**)—Fruit and seed development: immature fruit (**f**), ripe fruit (**g**), and seeds (**h**). Photo credits: Sinzinando Albuquerque-Lima (**a**,**c**,**f**), Zong-Xin Ren (**b**,**d**), Klaus Lunau (**e**), Bruno Henrique dos Santos Ferreira (**g**,**h**).

Vertebrates are central to the pollination ecology of Musaceae, with several species known to rely on birds and/or bats as pollen vectors [6,8,9]. This ecological relationship is associated with distinctive floral syndromes, including robust inflorescence architecture, tubular flowers, and nectar attributes (volume and sugar concentration), all of which facilitate vertebrate visitation [9,11,12,13]. Most of the reported cases of pollination in the family involve *Musa* species [6,8,9]. However, a particularly intriguing exception was reported by Liu et al. [7], who described insect pollination, primarily by bees and wasps, in *Musella lasiocarpa* (Franch.) C.Y. Wu ex H.W. Li, commonly known as the Golden Lotus (Figure 1 and Figure 2a,b).

This observation stands in contrast to the general patterns of vertebrate pollination in Musaceae and has shaped the prevailing view of *M. lasiocarpa* as an insect-pollinated species. Yet, the floral traits of the Golden Lotus, including its morphology, bright coloration, nectar properties, and lack of floral scent, are more consistent with adaptations to bird pollination [1]. Here, we present the first systematic reassessment of the pollination ecology of *M. lasiocarpa*, directly challenging the long-held assumption of insect pollination. By documenting new evidence of bird visitation involving multiple species, we demonstrate that the Golden Lotus fits squarely within the bird pollination phenotype typical of Musaceae.

The first systematic observations of pollination in the Golden Lotus were conducted between 2000 and 2001 by Liu et al. [7]. Their study reported pollination by several insect species, including bumblebees (*Bombus* spp. Figure 2c), honeybees (*Apis* spp. Figure 2d), and hornets *Vespa* spp. (Figure 2e). Although Liu et al. [7] noted the presence of sunbirds near inflorescences, they did not observe these birds actively visiting flowers.

It is important to consider methodological constraints when studying avian pollination. Unlike insects, which can often be approached closely, bird visitation requires careful observation at greater distances, typically using binoculars or long-lens cameras from no less than 10 m from the flowers. Such conditions may have limited the ability of Liu et al. [7] to detect bird-flower interactions.

While the pioneering data of Liu et al. [7] remain valuable, our expectations based on floral traits and subsequent observations, suggest that birds are frequent and ecologically important visitors to the flowers of *M. lasiocarpa*. This provided the motivation for our investigation, in which we present new data on the floral biology and avian visitors of the Golden Lotus, with methods designed to properly assess bird activity.

In addition to advancing our understanding of the reproductive biology of this emblematic species, our study reveals a broader overlooked role of birds in Asian pollination systems. Finally, we situate *M. lasiocarpa* within its cultural and historical context, underscoring its ecological, horticultural, and symbolic significance in Chinese traditions.

## 2. Material and Methods

The Golden Lotus (*M. lasiocarpa*) is a member of Musaceae (Zingiberales) native to southwestern China. Its original distribution is associated with the dry and hot river basins of the Upper Yangtze River in Yunnan and Sichuan provinces [15] (Figure 1). *Musella lasiocarpa* is a monoecious species that exhibits protogyny, in which female flowers (Figure 2a) are produced before male flowers (Figure 2b). This sequential sexual phase is clearly expressed in the inflorescence: flowers at the basal region are female, while those in the middle and terminal regions are male [7] (Figure 2). Flowering occurs year-round, making *M. lasiocarpa* an important and continuous resource for flower visitors and potential pollinators [7]. Pollinated female flowers produce fruits, and each fruit contains several hard, black seeds (Figure 2f–h).

**Figure 3:**
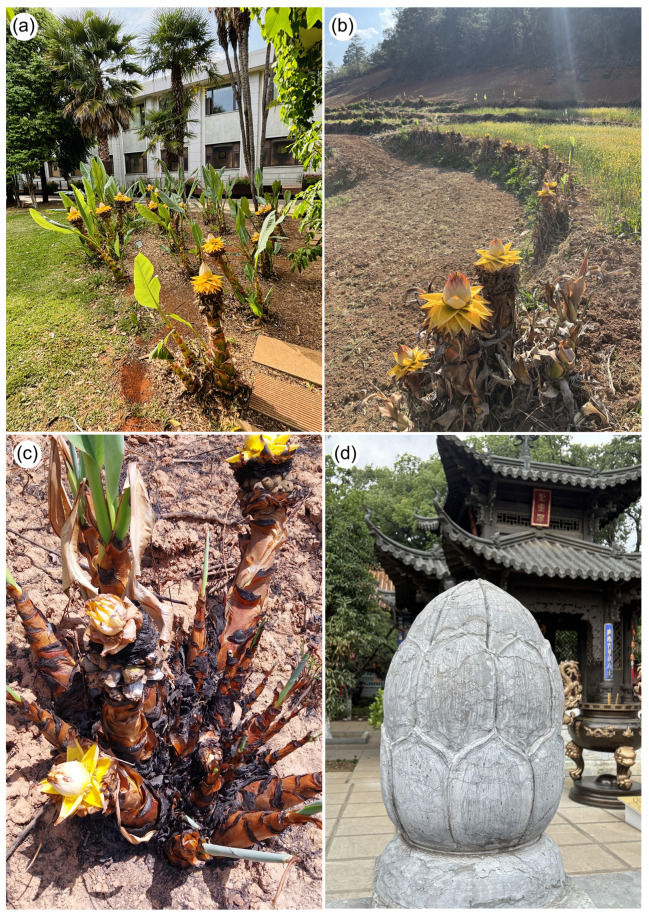
Selected populations of *M. lasiocarpa* and its symbolic representation in buildings in Kunming city, Yunnan, China. (**a**)—Population in Kunming Botanical Garden (KBG), Kunming, China, consisting of specimens planted for the 80th anniversary of the KBG. (**b**)—Population in Faketou Village, Aziying Town, in the mountains north of Kunming. Note that Golden Lotus plants can be used to separate areas with different crops. (**c**)—Population in the Zixishan region, Chuxiong city. Local management practices are evident, such as burning plants or removing dry leaves. Individual specimens are also planted to help stabilize soil on steep slopes. (**d**)—Stone wall structures referencing the Golden Lotus at Wenchang Temple, Heilongtan Park, Kunming, China. Photo credits: Sinzinando Albuquerque-Lima (**a**,**d**), Jeff Ollerton (**c**), and Zong-Xin Ren (**b**).

Today, wild populations of the Golden Lotus are difficult to locate or clearly define. However, due to its ornamental appeal, symbolic importance, and adaptability, *M. lasiocarpa* is widely cultivated in human-dominated landscapes (Figure 3a–d), including agricultural zones, villages, temples, and urban areas [16,17,18]. Many cultivated populations are found in agro-pastoral settings, especially in small farms managed by Yi and Han communities, who have played a central role in its spread and maintenance [16].

To test our expectations of potential bird pollination, we conducted observations on four populations of *M. lasiocarpa* for approximately 18 h between March and May 2024. The first population was located in Kunming Botanical Garden (KBG; Figure 3a), Kunming, Yunnan, China (25°08′27″ N, 102°44′27″ E, 2902 m a.s.l.); the second in the vicinity of Faketou Village (Figure 3b), Aziying Town in the mountains north of Kunming (25°20′24″ N, 102°46′02″ E, 2879 m a.s.l.); the third in the Huanglongqing area on the outskirts of Kunming city (25°05′24″ N, 102°48′18″ E, 2820 m a.s.l.); and the fourth in the Zixishan region (Figure 3c), Chuxiong city (25°04′23″ N, 101°25′20″ E, 2760 m a.s.l.). Notably, this last site corresponds to the same area where Liu et al. [7] carried out their insect-pollination study. In the Botanical Garden, plants occur both as isolated individuals and in groups, with our observations focusing on the largest groupings. The other populations allowed us to include non-urban contexts.

Bird visitation was observed by authors from a distance > 10 m away from the targeted plants using binoculars, and documented using a Nikon COOLPIX P100 camera (Tokyo, Japan). All observations were conducted from 07:00 to 17:30 throughout the day. Species were identified with the aid of specialized literature [19] and the Merlin Bird ID Database (The Cornell Lab, accessed in 2024; https://merlin.allaboutbirds.org/; accessed on 27 May 2024).

To complement our fieldwork, we also gathered records from citizen science platforms (iNaturalist, WeChat, TikTok; Table 1) following the approaches used by other researchers to use social media and citizen science to study birds [20,21,22]. We considered the advice by Spennemann [23] when using digital data and double checked every record to avoid redundancy among different platforms.

In 2019, images of inflorescence and flower color of two male stage inflorescences in KBG, as viewed by humans, in ultraviolet, and through the eyes of bees, were taken using standard and false-color photography methods [24]. In 2024, we randomly selected 15 flowers in KBG to measure floral morphology and nectar attributes. We bagged male flowers for 24 h using nylon bags after they opened. We picked flowers and measured floral corolla length and tube opening diameters by using a caliper with a resolution of 0.01 mm, and then measured the floral nectar attributes following Pyke et al. [25]. Nectar was withdrawn using graduated glass microcapillary tubes (10 μL, minicaps, accuracy: 0.5%, coefficient of variation: 1.0%; Hirschmann Laboratory, Eberstadt, Germany). Nectar volume was calculated from the height of nectar column in microcapillary tubes. Sugar concentration was measured with a manual refractometer (0 to 50% brix, Bellingham & Stanley Ltd., Tunbridge Wells, UK). To calculate sugar mass per unit volume (mg/μL), we used the equation Y = 0.00226 + 0.00937X + 0.0000585 × 2, where X is the sugar concentration (%) [25]. The total sugar content (mg) per flower was then determined by multiplying nectar volume (μL) by the sugar mass per unit volume (mg/μL).

## 3. Results

**Figure 4:**
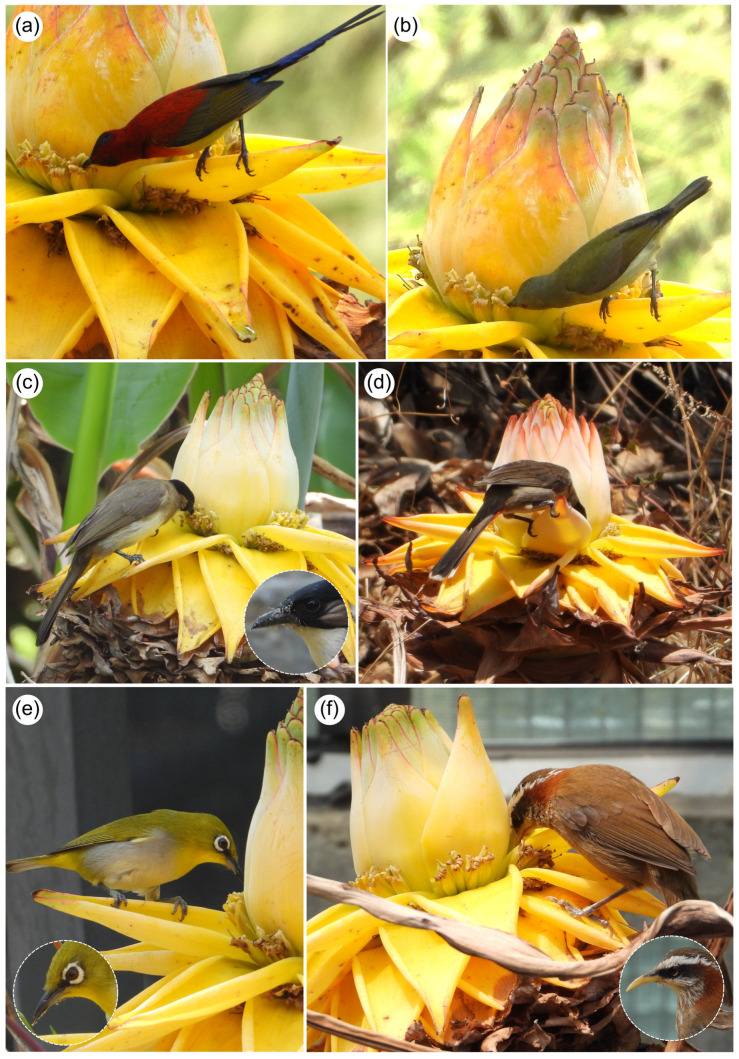
Flower-visiting birds of the Golden Lotus (*M. lasiocarpa*, Musaceae). (**a**,**b**)—Male (**a**) and female (**b**) of Mrs Gould’s Sunbird (*Aethopyga gouldiae* Vigors, 1831). (**c**)—Brown-breasted Bulbul (*Pycnonotus xanthorrhous* Anderson, 1869). (**d**)—Sooty-headed Bulbul (*Pycnonotus aurigaster* Vieillot, 1818). (**e**)—Indian White-Eye (*Zosterops palpebrosus* Temminck, 1824). (**f**)—Streak-breasted Scimitar Babbler (*Pomatorhinus ruficollis* Hodgson, 1836). Photo credit: Sinzinando Albuquerque-Lima.

A total of 12 bird species were confirmed as floral visitors of the Golden Lotus. Our direct observations recorded five species, while citizen science sources revealed an additional seven (Figure 4, Table 1). These species belong to five families: Muscicapidae (Old World flycatchers: 1 species), Nectariniidae (sunbirds and spiderhunters: 5), Pycnonotidae (bulbuls: 3), Timaliidae (scimitar-babblers: 1), and Zosteropidae (white-eyes: 2). All of these families are known floral visitors elsewhere [1].

During visits, all birds foraged for nectar (Figure 4a,b). One species, the Streak-breasted Scimitar-Babbler (*Pomatorhinus ruficollis*), also preyed on bees and other insects present on flowers (Figure 4f). Many birds carried visible pollen on their beaks and facial feathers (Figure 4c,e,f), strongly suggesting pollination. Male flowers received the majority of visits, although we also recorded Mrs Gould’s Sunbird (*Aethopyga gouldiae*) and the Indian White-eye (*Zosterops palpebrosus*) visiting female flowers. Bird visitation was found throughout the day, and there was no clear pattern to the timing of visits.

**Figure 5:**
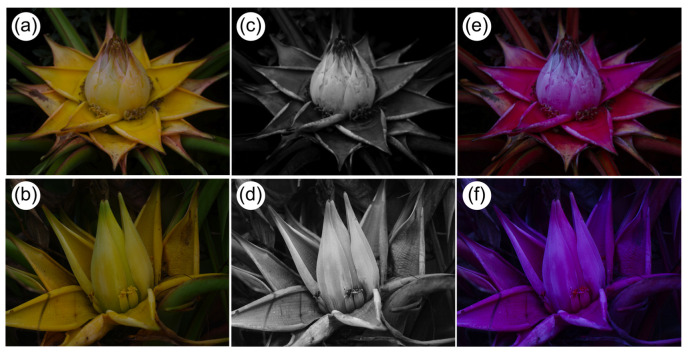
Variation in flower color of the Golden Lotus (*M. lasiocarpa*, Musaceae) as seen by humans (**a**,**b**), in ultraviolet (**c**,**d**), and through the eyes of bees (**e**,**f**) using false-color photography. Note that only the pollen of male flowers reflects ultraviolet light. Photo credit: Klaus Lunau. Photos were captured on 15 August 2019 in KBG.

Based on false-color photography, the inflorescence of *M. lasiocarpa* displays a visual signal with little contrast and no UV reflection, except UV absorption in pollen of male flowers (Figure 5). The tubular corolla length of male flower was 27.1 ± 0.43 mm (Mean ± SE, N = 15), the opening diameter was 1.2 ± 0.40 mm (N = 15). Each male flower produced a large amount of nectar reaching 10.6 ± 2.07 μL (N = 15) in volume. The sugar concentration is low, 12.2 ± 1.74% with a sugar mass of 1.7 ± 0.37 mg (N = 15).

## 4. Discussion

### 4.1. Evidence of Bird Visitation and Putative Pollination in Golden Lotus

The diversity of avian visitors to flowers clearly demonstrates that *M. lasiocarpa* is potentially pollinated by birds, in contrast to the earlier insect-focused account of Liu et al. [7]. The evidence is supported not only by the constancy of bird visitation, but also by floral traits characteristic of plants with bird pollinated flowers. Robust bracts, typical of Musaceae, provide landing platforms for birds and bats [8,26,27], and we consistently observed birds using them in *M. lasiocarpa* (Figure 4). The golden bracts are also highly conspicuous (Figure 5a) and, based on false-color photography [24], display a visual signal aligned with bird pollination: little contrast for bees, and no UV reflection, except in pollen of male flowers (Figure 5) [28]. A similar visual pattern occurs in some *Musa* species pollinated by bats [29]. Nectar attributes further reinforce this syndrome, with flowers producing large volumes (about 10 µL) per flower of dilute nectar (12% sugar concentration), typical of bird-pollinated plants [7,13,30].

Most bird visitors we recorded are established nectar foragers and pollinators of other plant species [1,31]. The five sunbird species (Nectariniidae) are especially noteworthy (Figure 4a,b), as this family is specialized for nectar feeding [1,2,32] and widely recognized as pollinators (or nectar thieves) in Africa and Asia [1,33,34,35,36,37,38,39]. Their narrow, curved bills are well adapted to the tubular flowers of *M. lasiocarpa*, as in other Musaceae species [9].

In addition to sunbirds, generalist passerine birds are important visitors to *M. lasiocarpa*. Generalist bird pollination also occurs in other culturally significant Chinese plants, reinforcing the broader ecological importance of ornithophily. For instance, *Rhododendron floccigerum* and *R. arboreum* (Ericaceae) [40,41], tea plants such as *Camellia petelotii* and *C. pubipetala* (Theaceae) [34,42], loquat (*Eriobotrya japonica*, Rosaceae) [43], and *Rhodoleia championii* (Hamamelidaceae) [44] all rely on birds for pollination. In this study, we did not quantify the relative contribution of birds to the reproductive success of *M. lasiocarpa*, though the evidence strongly indicates that birds may play a major role in its pollination. This is supported by both the floral traits of the species and the large number and diversity of avian visitors documented, and their behaviors. At the same time, our findings suggest that *M. lasiocarpa* may operate under a bimodal pollination system, in which both birds and insects (mainly bees) contribute to pollen transfer. Such dual systems have been described in several other families, including Apocynaceae, Bromeliaceae, and Iridaceae [45,46,47,48].

Although we have scientifically documented evidence of birds visiting the flowers of the Golden Lotus, familiarity with this interaction is apparently widespread as traditional knowledge in many rural communities in the Yunnan region. Local knowledge further supports our findings. Farmers in Faketou (population 2) and Zixishan (population 4) consistently reported bird visitation to Golden Lotus flowers for nectar. Such orally transmitted information illustrates the value of traditional knowledge in pollination ecology, which can inform biodiversity management [49] and, in some regions, provides insights into interactions predating European colonization [1].

Our study reinforces the importance of observations of the natural history of plant-pollinator interactions. Natural history is a descriptive approach to report on different aspects of the natural world, and in recent years there has been a recovery and increase in such publications, highlighting the importance of field observations to understanding aspects of pollination [50]. We thus confirm bird visitation and putative pollination in *M. lasiocarpa* for the first time, integrating natural history observations, citizen science data, and traditional knowledge. More broadly, it highlights the growing importance of natural history approaches in pollination ecology. In recent years, such descriptive work has been increasingly recognized, for example in the *Journal of Pollination Ecology*, and in dedicated sections in journals like *Ecology* (The Scientific Naturalist), *Biotropica* (Natural History Field Notes), *Ecology and Evolution* (Nature Notes), *Plants People Planet* (Flora Obscura), and in Special Issues such as “Ecology and evolution of plant-pollinator interactions: the importance of natural history” in *Flora* [50]. In the case of the Golden Lotus, only by revisiting earlier assumptions and conducting new natural history observations was it possible to reveal the putative role of birds as an additional, and possibly primary, guild of pollinators.

### 4.2. Golden Lotus as a Key Plant Resource for Animals and Humans

The Golden Lotus holds remarkable ethnobotanical significance, with medicinal, nutritional, cultural and religious uses [16,51]. Its pseudostem is consumed as a wild vegetable, while bracts and flowers are used in medicinal infusions by some ethnic minorities in southwestern China. The vegetative parts are also employed as fodder for pigs [16,17] (Figure 2). Beyond food and medicine, *M. lasiocarpa* is used as raw material for handicrafts: the midrib of dry leaves is processed into ropes, clothing items, and furniture such as chairs [16].

The Chinese name of *M. lasiocarpa* 地涌金莲 [di yǒng jīn lián], meaning “golden lotus that grows from the ground”, reflects both its striking appearance and cultural symbolism. In addition to its practical uses, it is widely valued for ornamental planting and religious significance [51] (Figure 3). Its current distribution is likely influenced by its role in landscaping; for example, 80 specimens were planted in Kunming Botanical Garden (KBG) to commemorate its 80th anniversary (Figure 3a).

**Figure 6:**
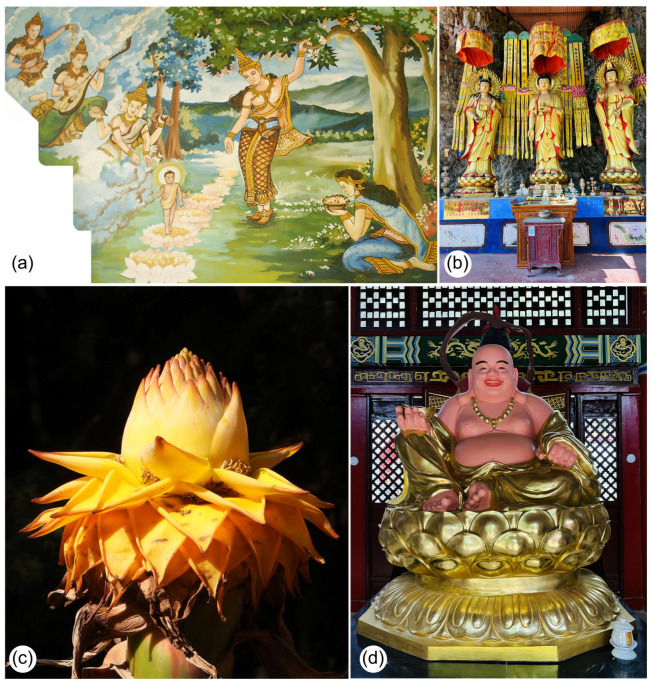
Representations of the Golden Lotus (*M. lasiocarpa*) in Buddhist culture and philosophy. (**a**)—Painting from a Buddhist temple in Laos depicting the birth of Buddha. According to legend, he took seven steps at birth and proclaimed that he would end suffering and attain supreme enlightenment. The seven steps are taken on seven Golden Lotus flowers (CC BY-SA 3.0: https://en.wikipedia.org/wiki/Buddhahood#/media/File:Birth_of_Buddha_at_Lumbini.jpg; accessed on 27 May 2024). (**b**)—Representation of Guanyinpusa (a female form of the Buddha) in Baoziqing, Kunming, China. Three female figures are shown standing under the Golden Lotus. (**c**)—Inflorescence of the Golden Lotus (*M. lasiocarpa*). (**d**)—Representation of Buddha seated on a Golden Lotus throne in a temple in Fengzeyuan Park, Kunming, China. *M. lasiocarpa* is considered one of the six flowers of Buddhism; the golden bracts and inflorescence shape symbolize the throne (open bracts) and the Buddha (overlapping bracts). Photo credits: Sinzinando Albuquerque-Lima (**b**–**d**).

**Figure 7:**
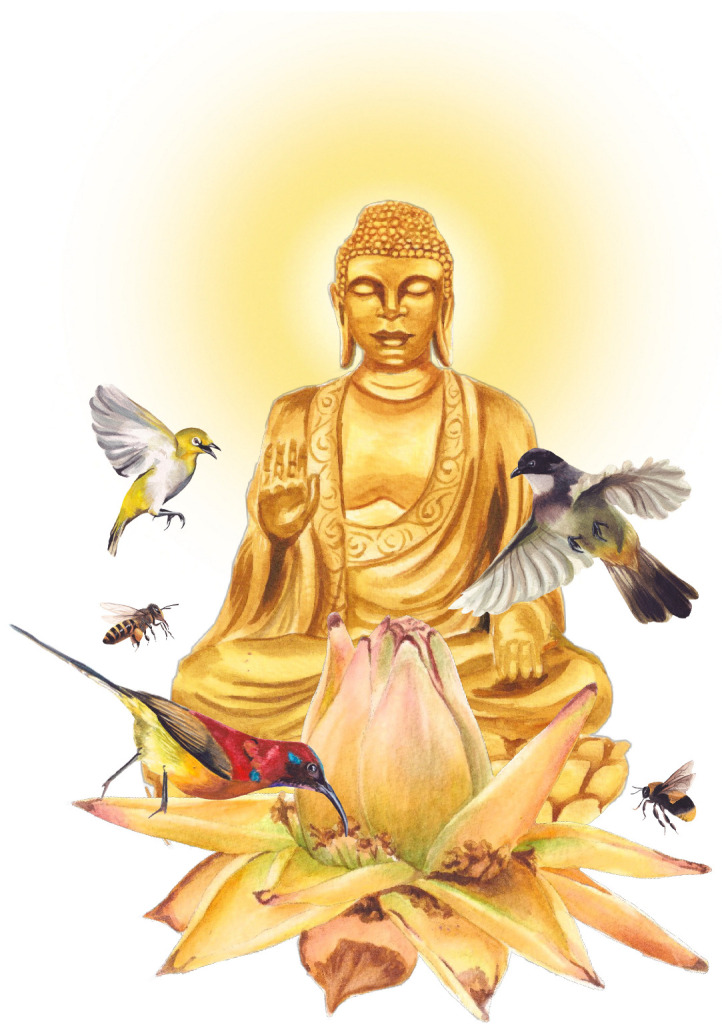
Illustration of the Golden Lotus (*M. lasiocarpa*, Musaceae), a culturally and spiritually significant plant in Buddhism, shown alongside its floral visitors and pollinators. Inspired by the *Dhammapada* (Minor Collection, Sayings of the Dhamma 44–59, Flowers): “A bee takes the nectar and moves on, doing no damage to the flower’s beauty and fragrance; and that’s how a sage should walk in the village.” This symbolism emphasizes the ecological importance of the species for pollinators and its significance in Buddhist tradition, reminding us that, like the sage, scientists should approach nature with respect and careful observation.

Culturally, the Golden Lotus is among the most important plants in Chinese Buddhism, often represented in temple gardens and religious iconography [51,52] (Figure 6). It is considered one of the six flowers of Buddhist philosophy and culture [53], with symbolic meaning linked to the Buddha (Siddhartha Gautama) [51]. Its golden bracts echo the frequent golden depictions of the Buddha and related figures (Figure 6b–d). The distinctive inflorescence shape also carries symbolic interpretation: the opened basal bracts represent the Buddha’s throne, while the unopened terminal bracts symbolize the Buddha himself (Figure 6c,d) [51]. Furthermore, *M. lasiocarpa* is associated with Guanyinpusa, a female form of the Buddha (Figure 6b). It is worth noting that *Nelumbo nucifera* Gaertn. (Nelumbonaceae), known as the Sacred Lotus, is also one of the six Buddha flowers and holds an equally important symbolic role in Buddhist aesthetics. However, its meaning differs: while the Golden Lotus emphasizes sacred representation through form and color, the Sacred Lotus embodies purity and transcendence within Buddhist philosophy. Our finding of sunbirds visiting *M. lasiocarpa* flowers enriches the cultural significance of this plant, because sunbirds are also iconic in Chinese culture, especially in southwestern China (Figure 7) [54].

## 5. Conclusions

Our results confirm a strong interaction between birds and the Golden Lotus, a culturally significant species in China and one of the flowers embedded in Buddhist philosophy (Figure 7). Both field observations and citizen science data supported our expectations, highlighting the importance of integrating natural history approaches with participatory science for advancing our understanding of plant-pollinator systems.

To our knowledge, this is the first scientific study to provide evidence of bird pollination in *M. lasiocarpa*, overturning the long-standing assumption of exclusive insect pollination and revealing a broader ecological role of birds in the reproductive biology of this iconic plant. Our findings thus enrich the ecological and cultural significance of *M. lasiocarpa* with implications for both biodiversity conservation and horticultural practices.

## Figures and Tables

**Table 1:** Families and species of flower-visiting birds of Golden Lotus, *Musella lasiocarpa* (Musaceae). In the last column, we note the type of observation source.

Family and Bird Species	English Name	Chinese Name	Evidence *
**Muscicapidae**			
*Copsychus saularis* (Linnaeus, 1758)	Oriental Magpie-robin	鹊鸲	Citizen science
**Nectariniidae**			
*Aethopyga gouldiae* (Vigors, 1831)	Mrs Gould’s Sunbird	蓝喉太阳鸟	Citizen science /Our study
*Aethopyga latouchii* Slater, 1891	Fork-tailed Sunbird	叉尾太阳鸟	Citizen science
*Aethopyga siparaja* (Raffles, 1822)	Crimson Sunbird	黄腰太阳鸟	Citizen science
*Aethopyga vigorsii* (Sykes, 1832)	Western Crimson Sunbird	猩红太阳鸟	Citizen science
*Arachnothera longirostra* (Latham, 1790)	Little Spiderhunter	长嘴捕蛛鸟	Citizen science
**Pycnonotidae**			
*Ixos mcclellandii* (Horsfield, 1840)	Mountain Bulbul	绿翅短脚鹎	Citizen science
*Pycnonotus aurigaster* (Vieillot, 1818)	Sooty-headed Bulbul	白喉红臀鹎	Our study
*Pycnonotus xanthorrhous* (Anderson, 1869)	Brown-breasted Bulbul	黄臀鹎	Our study
**Timaliidae**			
*Pomatorhinus ruficollis* Hodgson, 1836	Streak-breasted Scimitar Babbler	棕颈钩嘴鹛	Our study
**Zosteropidae**			
*Zosterops japonicus* Temminck & Schlegel, 1845	Japanese White-Eye	暗绿绣眼鸟	Citizen science
*Zosterops palpebrosus* (Temminck, 1824)	Indian White-Eye	灰腹绣眼鸟	Our study

* Notes: Links to each of the citizen science records can be found in Table S1—Supplementary Materials.

## Data Availability

The data that support the findings of this study are available in this article and in the Supplementary Materials of this article.

## Supplementary Materials

The following supporting information can be downloaded at: https://www.mdpi.com/article/10.3390/plants14203157/s1, Table S1. Links to each of the citizen science online records.
