# Supplementary material for: Beyond Bees: Evidence of Bird Visitation and Putative Pollination in the Golden Lotus (Musella lasiocarpa)—One of the Six Buddhist Flowers—Through Field Surveys and Citizen Science
Source: Plants (Basel). 2025 Oct 14;14(20):3157. doi: 10.3390/plants14203157 (PMC12567529; doi:10.3390/plants14203157)
Supplement: Supplementary file 1 [file plants-14-03157-s001.zip › plants-3884037-supplementary.pdf]

Table S1: Links to each of the citizen science online records.

BIRD SPECIES	LINKS
Aethopyga gouldiae	https://www.douyin.com/note/7347909551355612450
Aethopyga gouldiae	https://www.clzg.cn/article/269068.html
Aethopyga gouldiae	https://www.inaturalist.org/observations/222001755
Aethopyga gouldiae	https://www.bilibili.com/video/BV1GA4m1N7Fp?vd_source=b1032914870f60dc45eabe 214d60a155
Aethopyga gouldiae	https://www.meipian.cn/2vk5l1xy
Aethopyga latouchii	https://k.51vv.com/v/2S8Ru1nu
Aethopyga latouchii	https://www.xiaohongshu.com/explore/62ac6ad3000000000102cbb6?app_platform=ios&app_version=8.29&author_share=1&share_from_user_hidden=true&type=normal&xhsshare=QQ&shareRedId=N0o2N0VGSD42NzUyOTgwNjczOTc0PEc8&apptime=1712043000
Aethopyga latouchii	https://www.bilibili.com/video/BV11G411f7JT?vd_source=b1032914870f60dc45eabe214d60a155
Aethopyga latouchii	https://weibo.com/1263585953/4737936467692397
Aethopyga latouchii	https://weibo.com/1908847260/4862511683470337
Aethopyga latouchii	https://article.xuexi.cn/articles/index.html?source=share&art_id=6732019187698750127&study_style_id=video_default&share_to=weibo&study_share_enable=1&study_comment_disable=0&item_id=6732019187698750127&s_trans=2890026942_&s_channel=6
Aethopyga latouchii	https://weibo.com/2493429182/4734186713517060
Aethopyga latouchii	https://weibo.com/2420627037/4730674639016346
Aethopyga latouchii	https://weibo.com/5642655201/4729860407427971
Aethopyga latouchii	https://weibo.com/2164650254/4447698306974235
Aethopyga latouchii	https://baijiahao.baidu.com/s?id=1730003440524332769&wfr=spider&for=pc
Zosterops japonicus	
Ixos mcclellandii	
Aethopyga siparaja	https://www.inaturalist.org/observations/197766518
Aethopyga vigorsii	https://xsbn.yunnan.cn/system/2023/12/21/032881778.shtml
Aethopyga vigorsii	https://www.jianshu.com/p/b2ee0d8d7b43
Arachnothera longirostra	https://www.inaturalist.org/observations/201672379
Copsychus saularis	https://www.xiaohongshu.com/explore/64d274b5000000000a01b4d0?app_platform=ios&app_version=8.29&author_share=1&share_from_user_hidden=true&type=normal&xhsshare=QQ&shareRedId=N0o2N0VGSD42NzUyOTgwNjczOTc0PEc8&apptime=1712043664
